# Role of 18F-FDG PET-CT in Pre-Operative Planning of Surgical Debridement in Chronic Osteomyelitis

**DOI:** 10.1007/s43465-022-00771-9

**Published:** 2022-11-19

**Authors:** Ahmed Elsheikh, Mostafa Elazazy, Mamdouh Elkaramany

**Affiliations:** 1 Orthopaedic Surgery Department, Faculty of Medicine, Benha University, Fareed Nada Street, Benha, 13511 Egypt; 2 Al Bank Al Ahly Hospital for Integrated Care, Cairo, Egypt

**Keywords:** Osteomyelitis, PET-CT, Pre-operative planning, Debridement, Resection

## Abstract

**Purpose:**

Osteomyelitis is a challenge in diagnosis and treatment. 18F-FDG PET-CT provides a non-invasive tool for diagnosing and localizing osteomyelitis with a sensitivity reaching 94% and specificity reaching 100%. We aimed to assess the agreement in identifying the geographic area of infected bone and planned resection on plain X-ray versus 18F-FDG PET-CT.

**Methods:**

Clinical photos and X-rays of ten osteomyelitis patients were shown to ten consultant surgeons; they were asked to draw the area of infection and extent of planned surgical debridement; data will be compared to 18F-FDG PET-CT results.

**Results:**

We tested the agreement between the surgeons in every parameter. Regarding height, there was poor agreement between surgeons. Regarding perimeter, the ten surgeons showed low-moderate agreement. The ten surgeons showed a low-moderate agreement for circularity. Results document the variability of assessment and judgement based on plain X-rays. In comparison to PET-CT, All parameters were significantly different in favour of ^18^F-FDG PET-CT over X-ray (P < 0.001).

**Conclusion:**

^18^F FDG PET-CT provides a three-dimensional tool for localizing the exact location of the infected bone and differentiating it from the normal bone. Thus, it could be beneficial in precise pre-operative planning and surgical debridement of chronic osteomyelitis.

## Introduction

Osteomyelitis is a challenge in diagnosis and treatment; it involves bone and bone marrow[1]. Cierny-Mader classification of chronic osteomyelitis (COM) in long bone describes the anatomical involvement into medullary, superficial, localized or diffuse[2]. There are different modalities for diagnosing and analysing osteomyelitis with variable sensitivities and specificities like roentgenogram (plain X-rays), computed tomography (CT), magnetic resonance imaging (MRI) and several types of bone scintigraphy[3].

Fluorine-18 fluorodeoxyglucose (^18^F-FDG) is a radiolabeled glucose analogue taken by actively dividing living cells through a cell membrane transporter. ^18^F-FDG Positron Emission tomography (PET) depends on the Warburg effect, in which leukocytes, especially granulocytes and macrophages, show high glucose consumption once activated in response to inflammation, infection, and accelerated division like tumours [4].

^18^F-FDG PET, when fused with CT, provides a non-invasive tool for diagnosing osteomyelitis and differentiating between pathological and insufficiency fractures[5]. Additionally, it provides high-quality three-dimensional images that can localize the infection[6]. Compared to other imaging modalities, ^18^F-FDG PET-CT Sensitivity reaches up to 94%, while specificity reaches 100%[7]. Recently ^18^F-FDG was fused with MRI to avoid the radiation hazards and showed promising results[5]. 

Adequate debridement remains the gold standard of the treatment of COM; hence, the surgical planning for the debridement is challenging. Debridement aims to remove all infected or necrotic soft tissue and bone even if recognized intraoperative until the appearance of scattered pinpoint bleeding, i.e. paprika sign[8]. It also decreases the bacterial load and allows host immunity and antibiotic coverage to halt ongoing infection[9]. Good treatment needs a multimodality approach which includes debridement, dead space management and wound coverage[10]. 

This study aims to quantify the graphical role of ^18^F-FDG PET-CT in pre-operative planning of chronic osteomyelitis by answering the questions about where to debride and how much bone they should resect. Additionally, we aimed to compare the geographic area of infected bone and possible resection on plain X-ray versus ^18^F-FDG PET-CT. X-ray is the basic and first-line investigation of COM and is also widely used in developing countries as the sole imaging modality for COM.

## Methodology

This prospective study was conducted on the clinical photos, X-rays and ^18^F-FDG PET-CT images of ten patients with chronic osteomyelitis. The study was conducted according to the ethical principles of the declaration of Helsinki. Informed consent was taken from all patients involved in the study.

The clinical evaluation of the study subjects included a detailed sheet of all patients, including the patient’s complete history and examination findings. Clinical photos were taken to show the infected area, sinus and skin condition. The radiological evaluation of all patients was done using plain anteroposterior and lateral plain X-ray views and ^18^F-FDG PET-CT.

The history, clinical examination findings, and the infected part’s clinical photos were presented individually to ten consultant surgeons (five specialized in limb reconstruction and five general orthopaedic consultants).

Anteroposterior and lateral X-ray images were shown to the consultants using image editing software (Adobe Inc., 2019. Adobe Photoshop). They were asked to draw the area of possible infection and the extent of planned surgical debridement after reading the entire history and reviewing the clinical photos.

Data including the length of the possible infected segment (planned resection), width, area, perimeter and circularity of the possible infected segment were collected for the anteroposterior and lateral X-rays. The first author (AE) did a similar analysis on ^18^F-FDG PET-CT images of the same patients.

Data management and statistical analysis were done using SPSS version 28 (IBM, Armonk, New York, United States). Quantitative data were summarised as medians and ranges. Categorical data were summarised as numbers and percentages. Inter-observer agreement was assessed between surgeons using intraclass correlation coefficient (ICC), and a two-way random model was used. X-ray and ^18^F-FDG PET-CT readings were compared using Wilcoxon signed ranks test. All statistical tests were two-sided. P values less than 0.05 were considered significant.

## Results

The median years of experience of the included surgeons were three years and ranged from 1.5 to 6 years. The median annual infected cases treated by any surgeon were 28 (range 5 to 100 cases). The most common preferred tool for diagnosis was X-ray and CT together (50%). MRI was requested in 20% of cases, while the least frequent modality was CT alone (10%).

## The Findings of the X-ray and the Agreement Between the Surgeons

The mean reading of the X-ray parameters (perimeter, height, width, area, and circulatory) of the ten surgeons were summarised in Table 1. Individual readings were used to produce a mean for every surgeon to facilitate satistical analysis.

**Table 1:** Surgeons’ X-ray mean and range readings for all infected areas in the ten cases

	Surgeon 1		Surgeon 2		Surgeon 3		Surgeon 4		Surgeon 5		Surgeon 6		Surgeon 7		Surgeon 8		Surgeon 9		Surgeon 10
Perimeter	34.19		23.65		53.7		39.2		38.8		37.1		46.41		25		38		30
	(21.8—65.4)		(3.1—58.1)		(29.5—76.5)		(18.7—56.9)		(7.2—71.04)		(6.8—59.3)		(30.4—81.2)		(20—55)		(15.1—63.2)		(8.55—61.1)
Height	13.45		8.85		22.6		15.75		15.65		10.4		17.56		8.35		14.15		10.7
	(7.9—24.6)		(1.2—22.5)		(10.9—32.04)		(6.7—24.5)		(2.7—29.04)		(2.5—24.7)		(11.09—33.6)		(6.3—21.8)		(6.5—25.9)		(3.1—18.9)
Width	5.1		4.45		6.1		5.17		5.9		4.8		6.2		4.87		5.26		4.75
	(3.06—23.7)		(0.64—17.5)		(4.01—21.3)		(0.9—18.1)		(0.9—22.5)		(1.1—16.5)		(3.3—21.7)		(3—15.8)		(1.2—22.3)		(0.9—22.7)
Area	38.95		24.35		81.4		49.35		46.95		41.15		63.55		27.45		48.2		38.7
	(19.1—247.6)		(0.5—132.3)		(24.4—223.09)		(9.02—109.8)		(2.2—228.9)		(2.05—140.3)		(27.7—195.2)		(16.3—94.8)		(5.4—238.4)		(2.9—216.8)
Circularity	0.46		0.55		0.33		0.37		0.36		0.485		0.35		0.56		0.41		0.515
	(0.35—0.72)		(0.28—0.74)		(0.24—0.75)		(0.2—0.79)		(0.29—0.77)		(0.29—0.73)		(0.25—0.82)		(0.33—0.74)		(0.28—0.75)		(0.301—0.72)

All numbers are in pixels. Data were presented as medians and ranges

We tested the agreement between the surgeons in every parameter. Regarding height, there was a poor agreement between surgeons (ICC = 0.460, 95% CI = 0.266 – 0.685). Regarding perimeter, the ten surgeons showed a low-moderate agreement (ICC = 0.511, 95% CI = 0.307–0.732). The ten surgeons showed a low-moderate agreement for circularity (ICC = 0.577 & 95% CI = 0.392 – 0.770). Regarding area, a high moderate agreement was noted (ICC = 0.745, 95% CI = 0.583 – 0.876). An excellent agreement in width was reported (ICC = 0.913, 95% CI = 0.844 – 0.961). (Table 2).

**Table 2:** Agreement between surgeons’ X-ray readings

	Inter-observer agreement	
	ICC	95% CI
Height	0.460	0.266–0.685
Perimeter	0.511	0.307–0.732
Circularity	0.577	0.392–0.770
Area	0.745	0.583–0.876
Width	0.913	0.844–0.961

ICC: Interclass correlation coefficient

95% CI: 95% confidence interval

## Comparison Between the X-ray Parameters and the ^18^F-FDG PET-CT Readings

All parameters except circularity were significantly higher in X-ray than in the ^18^F-FDG PET-CT (*p* < 0.001). Circularity was significantly lower in X-ray than in the ^18^F-FDG PET-CT (*p* < 0.001).

The median perimeter reading was significantly higher in X-ray (36.2) than in ^18^F-FDG PET-CT (8.55) (*p* < 0.001). Also, the median height and width readings were significantly higher in x-ray (12.6 and 5.5, respectively) than in ^18^F-FDG PET-CT (2.95 and 2.05, respectively) (*p* < 0.001 for each). In addition, the median area reading was significantly higher in x-ray reading (44.1) than in ^18^F-FDG PET-CT (3.85) (*p* < 0.001).

The median circularity reading was significantly lower in X-ray (0.5) than ^18^F-FDG PET-CT (0.67) (*p* < 0.001) (Table 3). There was no correlation between any parameter and the years of experience ( *p *> 0.05) nor the number of cases per year (*p* > 0.05).

**Table 3:** Surgeon’s X-ray and 18F-FDG PET-CT readings of the infected areas

		Readings		
		X-ray	^18^F-FDG PET-CT	*P*-value
Perimeter	Median (range)	36.2 (11.3–59.7)	8.55 (5.3–23.1)	< 0.001*
Height	Median (range)	12.6 (4.7–23.8)	2.95 (1.8–6.7)	< 0.001*
Width	Median (range)	5.5 (1–20.2)	2.05 (1.3–7.4)	< 0.001*
Area	Median (range)	44.1 (4.1–182.6)	3.85 (1.3–30.4)	< 0.001*
Circularity	Median (range)	0.5 (0.3–0.7)	0.67 (0.49–0.7)	< 0.001*

Wilcoxon signed ranks test was used

*Significant

## Discussion

The pre-operative planning for osteomyelitis debridement surgery based on the X-ray analysis alone would not be the best modality. Although expected, this study represents the first to calculate and test the agreement between different observers; most parameters had a poor agreement; the width was not a problem as all surgeons planned to resect an entire bony segment. ^18^F-FDG PET-CT offered a precise and accurate tool to localize COM and plan the proper debridement plan.

Excision of all infected and necrotic bone is mandatory in surgical debridement of COM. However, it is not well known how to determine excision margins. The most commonly used technique is to debride the infected bone via extra-periosteal exposure, as stripping of periosteum adds more vascular compromise, resulting in iatrogenic sequestrum. The sequestrum must be detected and excised. A high-speed burr with continuous irrigation with saline could be used to decrease thermal necrosis[8].

During debridement, observation of bone should be done until the appearance of scattered pinpoint bony bleeding, referred to as the “paprika sign”, which is characteristic of viable bone and can be used to differentiate necrotic bone from viable one. In intramedullary COM, intramedullary reaming and canal irrigation are sufficient[11]. However, medullary reaming is not preferred if the infection is located at metaphysis or pre-operative x-rays show endosteal scalloping. Instead, debridement could be done by creating a cortical window that gives access to the medullary canal[12].

The level of surgical excision could be wide, marginal or intralesional. Wide excision means that all infected and necrotic bone is resected with a safety margin of 5 mm or more. All infected and necrotic bone in marginal excision is resected with less than 5 mm of clearance. Intralesional resection could be done by debulking infected tissue and removing small sequestrum, pus drainage, and lavage. Walenkamp et al. stated that there is a gradual transition between dead and viable bone [13].

Simpson et al. recommend that the type of the host would not affect the outcome of surgical resection if we do wide excision and that the recurrence rate with marginal excision in type B hosts is 50%, while in type A host is 0%[14]. They found partly necrotic and partly viable bone in the samples collected. Type A hosts may be able to resorb a microscopic amount of non-viable bone compared to type B hosts, which explains the difference between rates of recurrence between different hosts type [14].

The priority in planning for surgical debridement of osteomyelitis bone is to excise the whole infected bone and accurately determine the resection level to limit the need for serial debridement. Additionally, reaching out to healthy bleeding bone is mandatory to achieve union. Surgical planning and debridement of osteomyelitis depend on the surgeon’s experience to determine the resection level and differentiate between viable and necrotic bone. However, no method is described to distinguish infected bone from non-infected bone during the surgery.

Radiological assessment via X-rays, CT, and MRI provides different tools to localize the infection accurately and choose the best excision type and margin[3]. Nevertheless, these modalities could differentiate viable versus non-viable bone. Bone scintigraphy, in all forms, provides a functional test that locates actively metabolizing cells of active infection rather than viable bone[4]. This specific advantage makes scintigraphy a live streaming detector of osteomyelitis.

Bone scintigraphy and aforementioned radiological methods, except MRI, carry a radiation hazard[15]. Although radiation exposure quantification is complex and subject to many variables, CT Abdomen and pelvis exposes the patient to 10 millisieverts (mSv), while PET CT has been attributed to an effective dose of 25 to 30 mSv[16–18]. Studies have described some techniques and recommendations to decrease radiation exposure while doing hybrid nuclear imaging, with an estimated 32% reduction of radiation [15, 19].

Radiation exposure has been linked to radiation-induced cancers; however, a single exposure to PET-CT has a risk much lower than the natural risk of cancers[20]. The situation differs from repeated exams in cancer patients, where cumulative radiation dose adds a more considerable risk[21]. Therefore, Hybrid PET MRI has been investigated as an alternative, which provides high accuracy and decreases radiation exposure by up to 79% compared to PET-CT [21, 22]. On the contrary, Hulse et al. highlighted the superiority of PET-CT over PET MRI in accurate delineation, localization of infection and bone demarcation [23].

MRI has been an established method to detect and localize osteomyelitis, and its role in chronic osteomyelitis has been reported. Although MRI has a high sensitivity in COM detection, it has a modest graphical ability. Sequestrum appears black in all sequences, with increased uptake around. Defined borders are lacking, and the tendency to give false positive extensions is high[24, 25].

The choice to do PET-CT in this cohort resulted from the complexity of the cases (All were post-traumatic osteomyelitis, with a median duration of 22 months of active infection and a median of three failed interventions before the presentation. Lastly, half of them had metal inside). PET-CT was clinically justified on these bases, aiming to provide an accurate surgical plan of debridement[24, 26]. Demirev et al. found comparable accuracy between MRI and PET-CT in diagnosing OM. However, we focus on the pre-operative planning stage after establishing COM diagnosis (All had confirmatory clinical signs of OM)[26].

One study has highlighted the role of PET CT in pre-operative planning for COM. Christersson et al. analysed 8 patients with long-standing osteomyelitis, all had two types of radioisotope scan with few days apart. One is 18F-natrium-fluoride (NaF) PET detects the viability of the bone (cold spot), and the second is 18F-fluorodeoxyglucose (FDG) PET-CT which detects infection and inflammation (hot spot). They aimed to localize the infection precisely and reported a negative PET-CT scan after 12 months of surgical debridement. We agree with the author's view about the value of PET-CT in surgical planning; this could be done with the single standard 18F-FDG PET-CT. Also, the justification for repeating PET-CT after 12 months in clinically-well patients could be questionable[27].

In this study, we compared the value of using X-ray and ^18^F-FDG PET-CT in pre-operative planning for diagnosing the infected area; there was poor agreement between surgeons regarding the height (length) of the infected area depending on the X-ray alone. Results showed a high tendency to resect a more extensive segment of bone than the actual extent of osteomyelitis. This finding highlights the little value of X-ray alone in determining the infected segment.

The surgeon’s experience level has been proven to correlate with the rate of complications in joint replacement surgery [28]. Nevertheless, the surgical volume of the hospital is deemed to affect the outcomes of arthroplasty for femoral neck fracture[29]. Both studies were not related to the treatment of osteomyelitis, but we could argue that similar trends will be evident in these surgeries. Surgeons included in the study had a median of three years of experience. We could identify an extensive outline of infection drawn on the X-rays, with no comparable group of senior consultants.

The impact of poor localization of osteomyelitis could be devastating. Over-resection would lengthen the treatment duration, mandate more complex surgeries and increase the complications. Additionally, the patient could have more distress and psychological impact from prolonged treatment. On the other hand, under-resection would increase the recurrence rate and the number of surgeries which negatively affect the patients in all aspects.

Hence,^18^F-FDG PET-CT, which offers a precise geographical outline of the infected area (either viable or not), could be analysed in axial, coronal and sagittal planes, presents an objective method of osteomyelitis analysis and three-dimensional pre-operative planning, independent of the surgeon’s prediction or experience.

## Limitations

There are several limitations in our study, the number of patients of small. Despite having a high number of patients with osteomyelitis, we could not include more; the time and effort offered by the colleague consultants would be much more. Also, we could not increase the number of observers (colleague consultants) due to the unfamiliar purpose and methodology of the study. We could not commit to a radiologist due to the different centres of scintigraphy, which could also add another weakness due to variable techniques. 18F-FDG PET-CT was analysed by an orthopaedic surgeon (AE); however, his analysis agreed with the reported findings by the radiologist.

## Conclusion

**Fig. 1:**
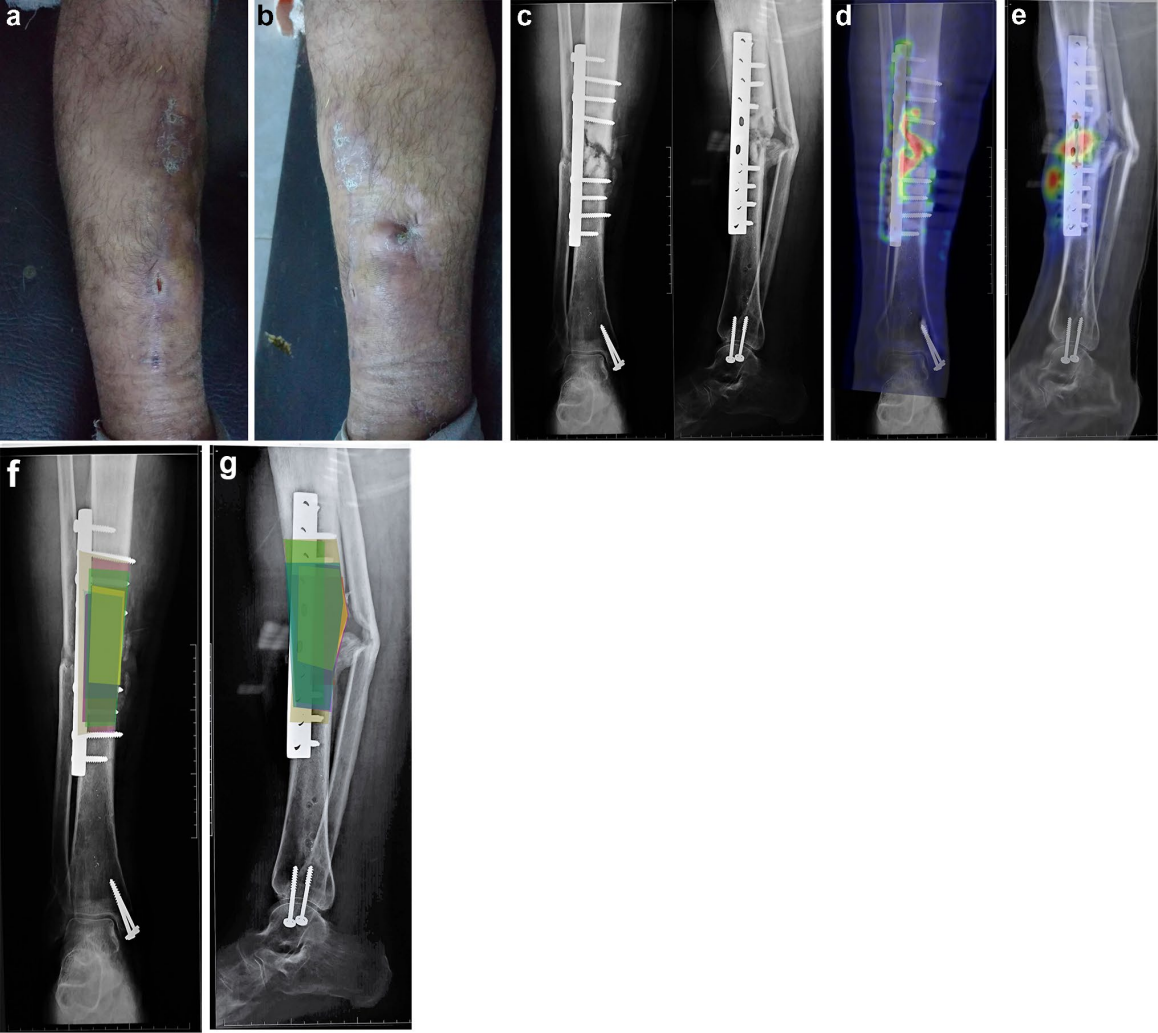
**a** Male patients, 30 years old, presented with postoperative osteomyelitis after fixation of Open fracture tibia after 9 months of infection development. The anterior aspect of the tibia shows a sinus. **b** Medial aspect of the leg shows a sinus. **c** Anteroposterior and lateral x-rays showed an infected non-united fractured tibia. **d**  18F-FDG PET-CT image merged with AP view of the x-ray, shows the clear demarkation of infection with the red colour referring to the highest uptake. **e** PET-CT image merged with lateral view of the x-ray, shows the clear demarkation of infection with the red colour referring to the highest uptake. **f** and **g** Anteroposterior and lateral x-ray with the layers drawn by the ten consultants, variability of the length can be appreciated

^18^F FDG PET-CT provides a three-dimensional tool for localizing the exact location of the infected bone and differentiating it from the normal bone. Thus, it could be beneficial in precise pre-operative planning and surgical debridement of chronic osteomyelitis (Fig. 1).

## Data Availability

All data generated or analysed during this study are included in this published article.
